# An Unusual Case of Petrous Apex Metastasis Revealed by Trigeminal and Abducens Nerve Palsies

**DOI:** 10.7759/cureus.83816

**Published:** 2025-05-09

**Authors:** Aiko Kishino, Kazuki Yamasaki, Syuji Yonekura, Toyoyuki Hanazawa

**Affiliations:** 1 Otorhinolaryngology, Sanmu Medical Center, Sanmu, JPN; 2 Otorhinolaryngology - Head and Neck Surgery, Chiba University, Chiba, JPN; 3 Otolaryngology/Head and Neck Oncology, Chiba University Graduate School of Medicine, Chiba, JPN

**Keywords:** abducens nerve palsy, carcinoma lung, ‏facial pain, fifth cranial nerve palsy, petrous apex

## Abstract

We present a case of an 81-year-old woman who developed facial pain, headache, hypesthesia, and diplopia. Although the diagnosis was initially challenging, further evaluation revealed a lesion in the petrous apex. Determining the nature of petrous apex lesions is often extremely difficult; however, due to imaging findings suggestive of malignancy or possible metastasis from another organ, whole-body CT was performed, ultimately leading to the diagnosis of metastatic disease from primary lung cancer.

Malignant tumors of the petrous apex are exceedingly rare and often asymptomatic in the early stages, contributing to their poor prognosis. Neurological symptoms, such as facial sensory impairment, may indicate the presence of a petrous apex tumor and should prompt further imaging evaluation. Moreover, because tumors in this region are frequently metastatic, a comprehensive systemic tumor search is essential for accurate diagnosis and appropriate management.

## Introduction

The petrous apex is a pyramid-shaped structure located in the medial portion of the temporal bone. Known pathologies in this region include inflammatory conditions, cholesterol granulomas, and cholesteatomas, while both primary and metastatic tumors are rare. Due to its deep anatomical location and proximity to critical neurovascular structures, petrous apex lesions are often asymptomatic or present with non-specific symptoms, making diagnosis challenging. Although cholesterol granulomas and cholesteatomas may exhibit relatively characteristic imaging features, neoplastic lesions can resemble inflammatory conditions, further complicating early detection [1].

While metastases to the temporal bone are uncommon, among the various regions of the temporal bone, the petrous apex is the most frequent site of metastasis [2]. The most common primary tumors that metastasize to the petrous apex include breast, lung, prostate, and kidney cancers [2].

In this report, we present a case of an 81-year-old woman with lung cancer metastasizing to the petrous apex. In this case, the diagnosis of a petrous apex tumor and identification of the primary malignancy were challenging due to the nonspecific nature of both the symptoms and imaging findings. This case highlights the diagnostic difficulty in differentiating petrous apex lesions when clinical and radiologic features are nonspecific.

## Case presentation

An 81-year-old woman presented to the internal medicine department of our hospital with a one-week history of headaches and right-sided facial pain. Her medical history included hypertension and hyperlipidemia; however, she had no history of smoking. Neurological examination revealed a right fifth cranial nerve (trigeminal nerve) palsy involving all three divisions, with no other abnormalities. On visual inspection and palpation, no skin rashes or other abnormalities were observed on the facial skin.

**Figure 1:**
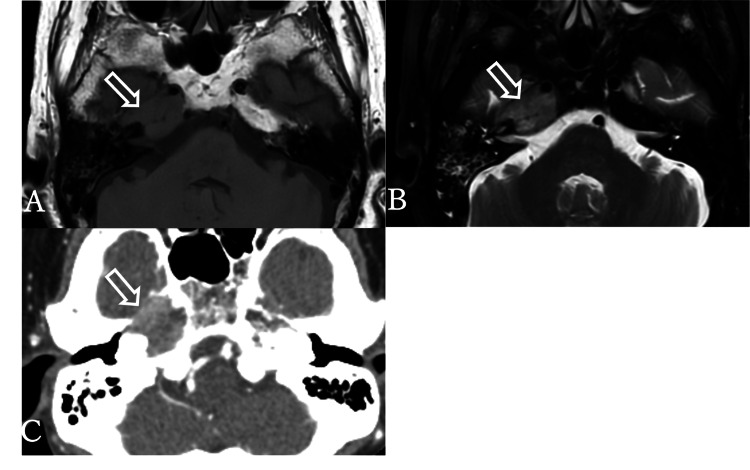
Axial MRI and CT of the temporal bone. (A) Axial T1-weighted MRI image. (B) Axial T2-weighted MRI image. A region in the right petrous apex appears isointense to brain parenchyma on T1-weighted imaging and iso- to mildly hyperintense on T2-weighted imaging. Additionally, an area in the ipsilateral mastoid, likely reflecting inflammation, is observed. (C) Contrast-enhanced CT scan performed for systemic evaluation. A tumorous lesion corresponding to the right petrous apex is identified, showing partial contrast enhancement. The lesion extends with bone destruction of the petrous bone.

Given the localized right facial pain and sensory impairment limited to the right trigeminal nerve distribution, the internist suspected herpes zoster and prescribed valacyclovir and pregabalin. The symptoms temporarily improved; however, despite good treatment adherence, the pain recurred one week later. She was subsequently referred to our otolaryngology department. Additionally, the patient developed new-onset diplopia, and right sixth cranial nerve (abducens nerve) palsy was confirmed. The coexistence of right trigeminal nerve palsy and right abducens nerve palsy prompted brain magnetic resonance imaging (MRI) to evaluate intracranial pathology. MRI revealed asymmetric signal changes in the right petrous bone with isointense shadows on T1-weighted images and slightly hyperintense shadows on T2-weighted images (Figures 1A, 1B). The initial differential diagnoses included inflammatory changes or tumors. However, the lesion extended beyond the petrous bone into the cavernous sinus surrounding the internal carotid artery, raising the suspicion of a tumor, particularly a metastatic lesion.

To further investigate the petrous apex lesions and identify systemic malignancies, contrast-enhanced whole-body computed tomography (CT) was performed. A tumorous lesion with enhancement and bone destruction was identified in the right petrous apex (Figure 1C).

**Figure 2:**
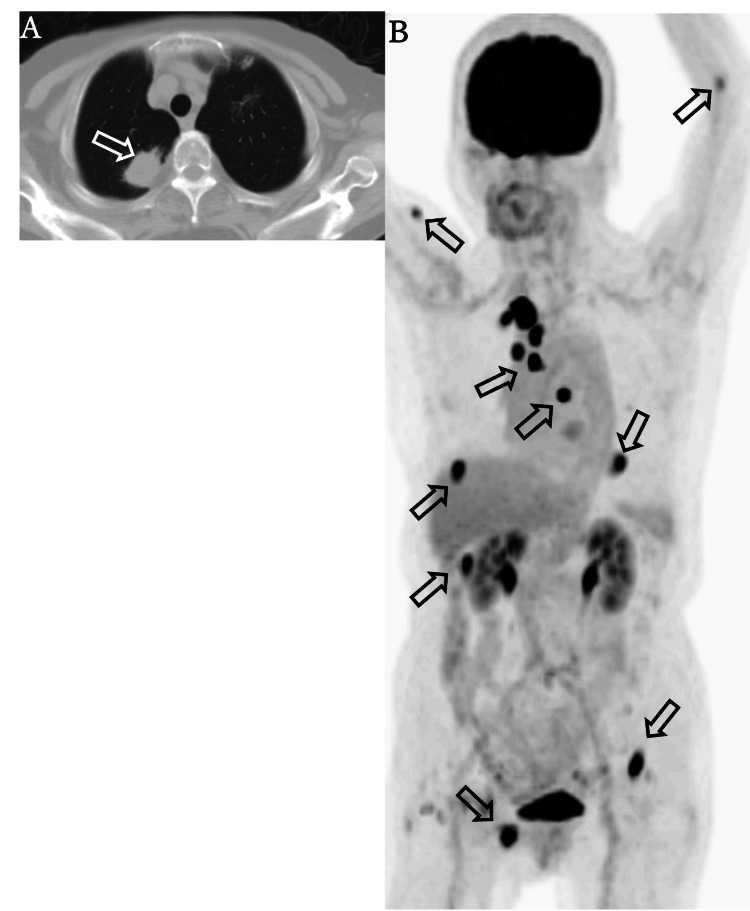
Chest CT (pulmonary window) and PET-CT scan. (A) A nodular shadow with pleural indentation is observed in the S1 segment of the right upper lung lobe, within the lung field striae. (B) Increased maximum standardized uptake value (SUVmax) accumulation is noted in the hilar lymph nodes (#3a, 4R), liver S8 region, left proximal radius, right pubis, myocardium, right kidney, right brachialis, left inguinal iliac region, and right 3rd-4th intercostal spaces, all considered metastatic lesions from lung cancer.

Additionally, a 31 mm mass was identified in the S1 segment of the right upper lung lobe (Figure 2A). Based on these findings, the lesion in the petrous apex was suspected to be a metastasis from primary lung cancer, and the patient was referred to the respiratory medicine department of a tertiary medical center. Subsequent PET-CT imaging demonstrated increased uptake in multiple regions, including the right petrous apex, hilar lymph nodes (#3a and 4R), a 10 mm lesion in segment S7 of the liver, a 19 mm lesion in the lateral aspect of the right kidney, as well as the left proximal radius, right pubis, myocardium, right kidney, right brachialis, left inguinal iliac region, and the right 3rd-4th intercostal spaces. Increased uptake was also observed in the right upper lung lobe (Figure 2B). These findings were consistent with primary lung cancer and widespread distant metastases.

Endobronchial ultrasound-guided transbronchial needle aspiration (EBUS-TBNA) of the lung tumor revealed negative staining for TTF-1 and napsin A mucin, and positive staining for p40 and CK5/6, leading to a diagnosis of non-small cell lung carcinoma (NSCLC) that was not otherwise specified (NOS).

Approximately 60 days had passed since her initial presentation to the internal medicine department at our hospital. Owing to the significant impairment in activities of daily living (ADL) caused by facial pain and headache, external radiation therapy (20 Gy) was urgently administered to the petrous apex.

**Figure 3:**
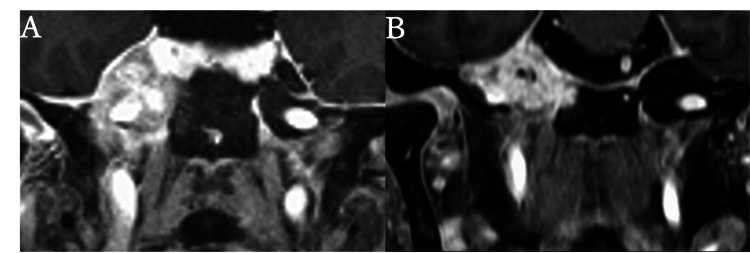
Contrast-enhanced MRI (coronal view) before and after treatment. (A) A contrast-enhancing tumor is observed occupying Meckel’s cavity. (B) The metastatic lesion in the right petrous bone shows an overall reduction in volume.

Post-radiation MRI revealed tumor shrinkage in the petrous apex (Figure 3), and the patient's headache, otalgia, and facial pain rapidly improved with the resolution of eye movement restriction.

The lung biopsy specimen was submitted for the Oncomine™ Dx Target Test, which detected an EGFR L858R mutation, leading to the initiation of osimertinib therapy.

One month after starting the osimertinib treatment, the patient remained stable with no observed adverse events.

## Discussion

The petrous apex is a pyramid-shaped structure that is located in the medial portion of the temporal bone. The lateral area adjacent to the petrous apex lies in the cavernous sinus, which contains the internal carotid artery, trigeminal nerve, facial nerve, and other neural structures. In addition, the Dorello’s canal carries the abducens nerve within the cavernous sinus. Owing to the anatomical localization of these nerves, petrous apex lesions often present initially with trigeminal nerve symptoms; as they progress, they may lead to various neurological deficits [3].

The petrous apex naturally exhibits asymmetry owing to variations in the development of mastoid air cells, even in normal cases. Physiological conditions such as bone marrow within the petrous apex and fluid accumulation are commonly observed, and asymptomatic encephaloceles extending from the Meckel’s cavity are also known to occur. These conditions do not require treatment, and although asymmetry may be present, they are commonly termed "Leave Me Alone" lesions, which complicate the differentiation between normal anatomical variations and pathological abnormalities.

Petrous apical lesions are rare but encompass a wide range of pathological conditions. Cholesterol granulomas are the most common, accounting for most petrous apex lesions. It forms because of chronic hemorrhage and the accumulation of its degradation products, characteristically presenting as hyperintense on both T1- and T2-weighted MRI images. Subsequently, cholesteatoma-related otitis media occurs in a small percentage of cases, more frequently in younger individuals, and typically appears hypointense on T1-weighted images and hyperintense on T2-weighted images.

By contrast, neoplastic lesions at the petrous apex are relatively rare. Primary tumors such as meningiomas and schwannomas have been reported in this region, although they are uncommon. Additionally, when lesions are present in the petrous apex, the possibility of inflammatory diseases, such as osteomyelitis or abscess formation, should be considered. These may present with an inflammatory spread pattern on MRI and can mimic neoplastic processes, necessitating careful differentiation [3].

Importantly, both metastatic and inflammatory lesions may appear hyperintense on T2-weighted images and hypointense on T1-weighted images. However, inflammatory lesions can exhibit a range of imaging characteristics, whereas the presence of osteolytic changes is more specifically indicative of bone metastasis and serves as a key distinguishing feature.

In our case, MRI revealed isointense signals on T1-weighted images and slightly hyperintense signals on T2-weighted images at the petrous apex, which were inconsistent with findings typically seen in cholesterol granulomas or cholesteatoma-related otitis media. CT imaging revealed a contrast-enhancing tumor with osteolytic destruction in the right petrous apex, corresponding to the area of abnormal signal seen on MRI. Additionally, a mass was detected in the right upper lung lobe, raising suspicion for a primary malignancy. The identification of bone destruction on CT was a key finding that supported the diagnosis of metastatic disease to the petrous apex from lung cancer.

Notably, osteolytic changes further reinforced the likelihood of a neoplastic process. Additionally, non-small cell lung adenocarcinoma is well known for its propensity to metastasize to the skeleton. Tsuya et al. reported that 30.4% of patients with non-small cell lung adenocarcinoma had skeletal metastases, and among them, approximately 60% presented with bone metastases at the time of initial diagnosis [4]. This supports the conclusion that the petrous apex lesion in our case was consistent with metastasis from non-small cell lung adenocarcinoma.

Although metastatic involvement of the temporal bone is uncommon, the petrous apex is the most frequently affected site for metastatic tumors within the temporal bone [2]. Patients with petrous apex lesions are often asymptomatic; however, as the disease progresses, they may develop headache, retro-orbital pain, or otalgia [5]. Petrous apical metastases are most commonly observed in patients aged 50-70 years. Breast cancer is the most common primary tumor that metastasizes to the petrous apex, followed by lung, prostate, and kidney cancers [2]. Moreover, a few case reports have described metastases from less common primary sites such as thyroid and rectal cancers. These reports help expand the differential diagnosis when evaluating petrous apex lesions [6,7]. The accepted explanation is that the bone is highly vascularized, and the bone marrow and trabeculae serve as a network for tumor seeding, making hematogenous metastasis more likely [8]. In addition, a venous network known as Batson’s plexus, which connects the perivertebral venous system, is thought to have potential communication with the venous structures of the temporal bone, further facilitating metastatic spread [1].

Metastatic tumors typically first affect the petrous apex and then spread to adjacent temporal bone structures via the osseous and perineural routes. These tumors often remain clinically silent until they progress significantly, and symptoms appear only when surrounding structures, such as the cranial nerves, internal carotid artery, or cavernous sinus, are affected. In addition, cases in which the petrous apex is involved in bone metastasis often present with multiple metastases at other locations. Consequently, the prognosis for temporal bone metastases is poor, with a five-year survival rate of only 30% [9].

As observed in this case, petrous apex metastases often lack specific clinical symptoms and exhibit nonspecific imaging findings, leading to their misinterpretation as an inflammatory process. This may result in the unnecessary administration of antibiotics or other medications, causing delays in reaching a definitive diagnosis by several months or even years [9]. Even when a tumor is suspected rather than inflammatory, distinguishing whether the petrous apex lesion is primary or metastatic based on symptoms alone is difficult, and predicting the primary site is equally challenging.

For a definitive diagnosis, depending on the tumor location, surgical approaches such as the middle fossa approach or the infralabyrinthine approach may be performed for histopathological examination. However, these procedures are highly invasive, carry a high risk of complications, and cannot be performed routinely [10]. In our case, we did not proceed with a biopsy of the petrous apex. Instead, based on imaging findings, including whole-body CT and PET-CT, which revealed multiple bone metastases, the lesion at the petrous apex was concluded to represent metastatic disease from lung cancer.

Notably, Henrich et al. reported that 24% of 249 autopsied patients with primary tumors outside the temporal bone had metastases to the temporal bone [9]. Furthermore, da Silva et al. reported that one of 18 cases of malignant tumors in the temporal bone was due to metastatic solid cancer [11]. These findings suggest that undiagnosed temporal bone metastases may be present in patients with cancer and that when a tumorous lesion is found in the temporal bone, the possibility of metastasis must be carefully considered.

Therefore, when a tumorous lesion is suspected in the temporal bone, a systemic tumor screening is essential. If a patient presents with cranial nerve dysfunction, CT or MRI of the head and neck should be performed at an early stage. Furthermore, if a lesion involving the petrous apex or other parts of the temporal bone is detected, whole-body CT or PET/CT should be performed, while considering the possibility of metastatic disease.

## Conclusions

Although diseases of the petrous apex often lack specific clinical symptoms or imaging findings, neoplastic lesions must be considered in the differential diagnosis. When a neoplastic lesion is suspected, metastatic disease should always be considered; however, obtaining a biopsy is often challenging. Therefore, when a petrous apex tumor is suspected, a comprehensive search for the primary lesion is essential.
